# Barriers and opportunities to oral health in Dutch-Moroccan children in the Netherlands: a narrative report

**DOI:** 10.1007/s40368-018-0367-3

**Published:** 2018-08-20

**Authors:** K. A. van Nes, J. S. J. Veerkamp, R. Reis

**Affiliations:** 1 Department of Cariology, Endodontology Pedodontology, Academic Centre for Dentistry Amsterdam (ACTA), University of Amsterdam and VU University Amsterdam, Gustav Mahlerlaan 3004, room 2N41, 1081 LA Amsterdam, The Netherlands; 2 Department of Public health and Primary Care, Leiden University Medical Centre (LUMC), Postbus 9600, 2300 RC Leiden, The Netherlands; 3 Amsterdam Institute for Social Science Research (AISSR), University of Amsterdam, Amsterdam, The Netherlands; 4 The Children’s Institute, University of Cape Town (UCT), Cape Town, South Africa

**Keywords:** The Netherlands, Culture, Dutch-Moroccan, Qualitative research, Children’s oral health, Knowledge

## Abstract

**Introduction:**

Previous studies showed that 5-year-old Dutch-Moroccan children had significantly higher dmft scores compared to Dutch children of the same age, even after correction for socio-economic status. The mechanisms underlying this difference are little understood.

**Aim:**

To explore cultural factors involved in poorer oral health of Dutch-Moroccan children by identifying knowledge, attitudes and behaviour of their mothers concerning their children’s oral health.

**Methods:**

In 2012 mothers of Dutch-Moroccan preschool children in two cities in the Netherlands were interviewed in two focus groups (n = 16) or individual semi-structured interviews (n = 13). Semi structured interviews were also c onducted with three oral health professionals, working with Dutch-Moroccan children, and one physician from an under-five-clinic. All interviews were voice recorded, transcribed and inductively coded. MAXQDA software was used for data analysis.

**Results:**

All mothers mentioned pain complaints, swelling and black front teeth as oral health problems in their children. Although mothers were aware that brushing teeth and reducing sugary snacks are effective preventative strategies, they did not sufficiently implement these measures. This was due to lack of brushing skills, insufficient awareness of the daily sugar intake of their children and their childrearing concerning these measures. Most mothers indicated they felt empowered in making dental care decisions.

**Conclusions:**

This research revealed the presence of knowledge on preventive strategies regarding their children’s oral health in Dutch-Moroccan mothers, but an inadequate implementation of these measures in their daily lives. Additional qualitative research is needed to gain deeper insight for broader exposure of values, knowledge and culture.

## Introduction

In the Netherlands, for very young children, caries is the main oral health disease. Caries results from high frequent sugar consumption; lack of oral hygiene and lack of use of fluoride (WHO 2007). These behaviours are influenced by numerous environmental, economic and social factors, such as lack of education (Verrips et al. 1992; Verrips et al. 1993b), lack of dental knowledge (Hooley et al. 2012), low oral health literacy (Miller et al. 2010; Divaris et al. 2011), low socio-economic status (De Vries 1989), gender (Verrips et al. 1993b), family functioning (Duijster et al. 2014), locus of control (Duijster et al. 2015b), and ineffective governmental strategies (De Vries 1989; Van Loveren and Van der Weijden 2000; Van Palenstein Helderman 2011). Given that low fluoride intake, more cariogenic bacteria, high frequency of sugar intake and poorer oral hygiene are observed in children from low socio-economic status and ethnic minority groups; worldwide these groups have increased caries risk (Burgersdijk and Truin 1997). In 2011, 66% of 5-year old children in the Netherlands were caries-free. The dmfs decrease since 2005 mainly occurred among children in families with higher socio-economic status (SES) (Abbink and Den Dekker 2013). As minority groups, in particular migrants from non-Western background, generally have lower SES this implies that children from these groups are still at greater risk for reduced oral health (Verrips et al. 1992, 1993a, b; Verrips and Kalsbeek 1993; Truin et al. 2007; Sabbah et al. 2009). In the Netherlands the major minority groups of non-Western immigrant background are Surinam, Turkish and Moroccan, while the latter is the main ethnic group in Amsterdam (CBS 2014).

Literature is not conclusive about the risk factors for caries development in these ethnic minority groups. Van der Tas et al. (2016) concluded that the odds for severe caries are significantly higher for children in Turkish, Surinam-Hindustan and Moroccan groups in the Netherlands. However, they found that socio-economic position and oral health behaviour could only partially explain these results. Vermaire (2013) also found that ethnicity itself is not a risk factor for the prevalence of dental caries. Although ethnicity of the mother was significantly associated with higher dmfs (De Vries 1989; Abbink and Den Dekker 2013), between minority groups no difference was found in the consumption of high sugar content foods (Verrips and Kalsbeek 1993). In research on the dental health status of different ethnic and cultural groups in the Netherlands, Verrips and Kalsbeek (1993) observed that: “*The mechanism underlying the differences in caries experience between various groups is still little understood*”. Brugman et al. (1998) found that Turkish and Moroccan parents in the Hague differed in their oral disease preventative behaviour. Although we know that different ethnic groups vary in types of self-care that are important for maintaining oral health, e.g. frequency of brushing, fluoride intake, and age of starting to brush baby teeth (Verrips et al.1993b), the differential causal pathways to reduced oral health for ethnic minority groups have not yet been established. Cultural factors that influence oral health behaviour need further exploration.

In the Netherlands, research on cultural determinants of health is complicated by problems of definition. Firstly, migrant status is officially determined by having at least one parent who was born outside of the Netherlands; this implies that third generation migrant children are registered as Dutch, which influences statistical analysis and hinders understanding the relationship between caries and cultural groups. Secondly, the sole focus on individual determinants and their association with caries and the neglect of social aspects of health care behaviour in former studies have obscured motivations of these low income families for dental care seeking. Thirdly, quantitative research among ethnic minorities may encounter problems in translation and distribution of questionnaires (Hoopman et al. 2009). Marmot (2005) states that major determinants in health are social, and suggests that the remedies must be social as well. Internationally, some qualitative research has been published discussing the social and cultural mechanisms in oral health in children. In 2007, Hilton et al. showed in a qualitative study the underlying norms and values of parents regarding their children’s oral health of four different ethnic groups within the United States of America. The worldwide notice for qualitative research on the patient perspective on oral health is visible in more recent studies. Perspectives of parents towards their children’s oral health have cbeen investigated qualitatively in the USA in Bangladesh immigrant families by Karasz et al. (2014) and in the UK by Aljafari et al. (2014) and Trubey et al. (2014). This exploratory study was in answer to the need for more qualitative studies on cultural variables and themes that may explain oral health disparities in children in the Netherlands.

**Fig. 1:**
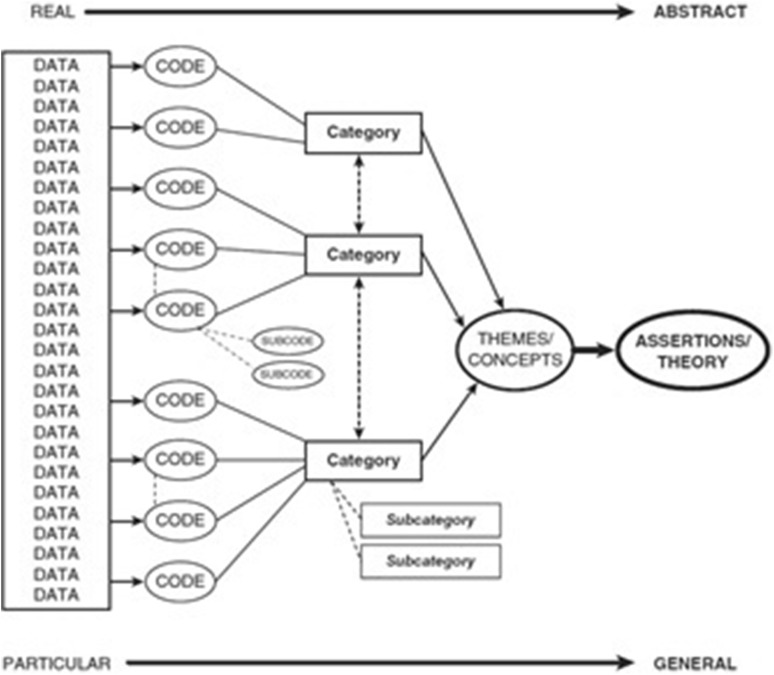
A streamline code to theory model for qualitative inquiry (Saldaña 2015)

The purpose of this research was to further explore underlying cultural mechanisms that determine causal pathways to reduced oral health of Dutch Moroccan children. To achieve this aim, this research focussed on mapping knowledge, attitudes, and practices of Dutch Moroccan mothers concerning their children’s oral health. This study focused on mothers, since they are usually involved in daily care practices. On the basis of the study findings a theoretical model was developed on Dutch Moroccan mothers perspectives on their children’s oral health (Fig. 1).

## Materials and methods

### Study design

**Fig. 2:**
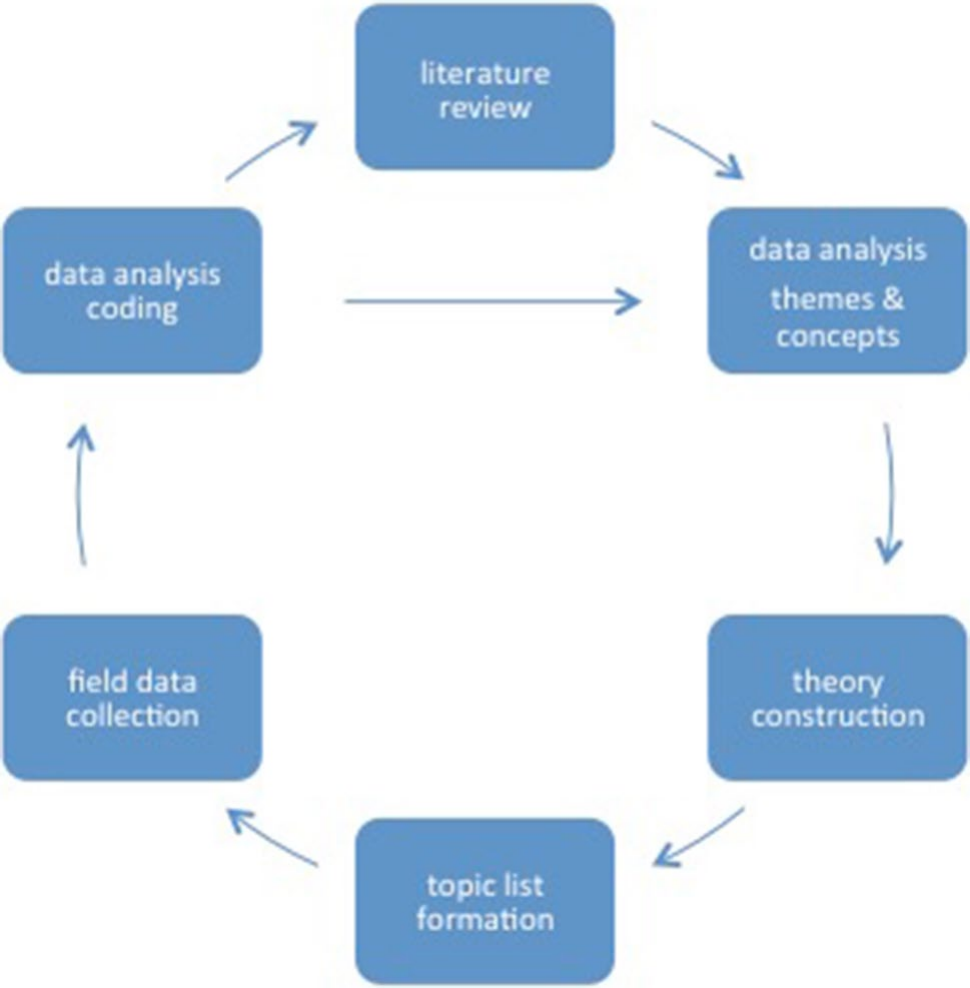
Iterative approach between theory, data gathering, analysing, reflection and adaptation of the topic list

The research was carried out between October 2011 and May 2013. The research was designed according to grounded theory research methods to develop a theoretical model for Dutch Moroccan mothers’ perspectives on their children’s oral health. A grounded theory is formed by the reciprocal relationship of data collection, analysis and theory (cf. Straus and Corbin 1990: 24). The approach was iterative, with a continuous cycle between theory, data gathering, analysing, reflection and adaptation of the topic-list (Fig. 2). Data was collected through literature search, focus group discussions (FGD) and semi structured interviews (SSI). Focus Group Discussion was chosen as the preferred methodology as it is suitable for exploration of social norms and stimulates discussion. Individual interviews were added to obtain more detailed and confidential information without group influences. Data was systematically coded and analysed with open and axial coding. Coding of the raw data was both deductive and inductive. The coding system with initial codes and sub-codes related to themes deductively derived from literature, was supplemented by inductively coded newly emerged themes from interviews data.

### Recruitment sampling

Since this study was a small-scale exploratory study, purposive sampling was used to obtain a sample with the various characteristics and distribution of the population. Participants were recruited via convenience and snowball sampling, in paediatric dental care, at elementary schools and primary schools in the Hague and Amsterdam (Ritchie et al. 2003). Dutch Moroccan mothers from the 1st, 2nd and 3rd generation with a child in the age group of 0–5 years old were included in the research. Mothers were informed verbally and in writing by the researcher about the scientific purpose of the research and the anonymous and confidential data processing, including the use of pseudonyms, to provide a just ethical procedure. After being informed about the study, mothers were asked to participate. For those willing to participate, an appointment was scheduled at school after the mothers brought their child to class or before they picked up the child from school. The recruitment ended when data saturation was obtained which was when new interviews yielded no new information. No compensation was given to the mothers for their participation.

### Data collection and analysis

**Fig. 3:**
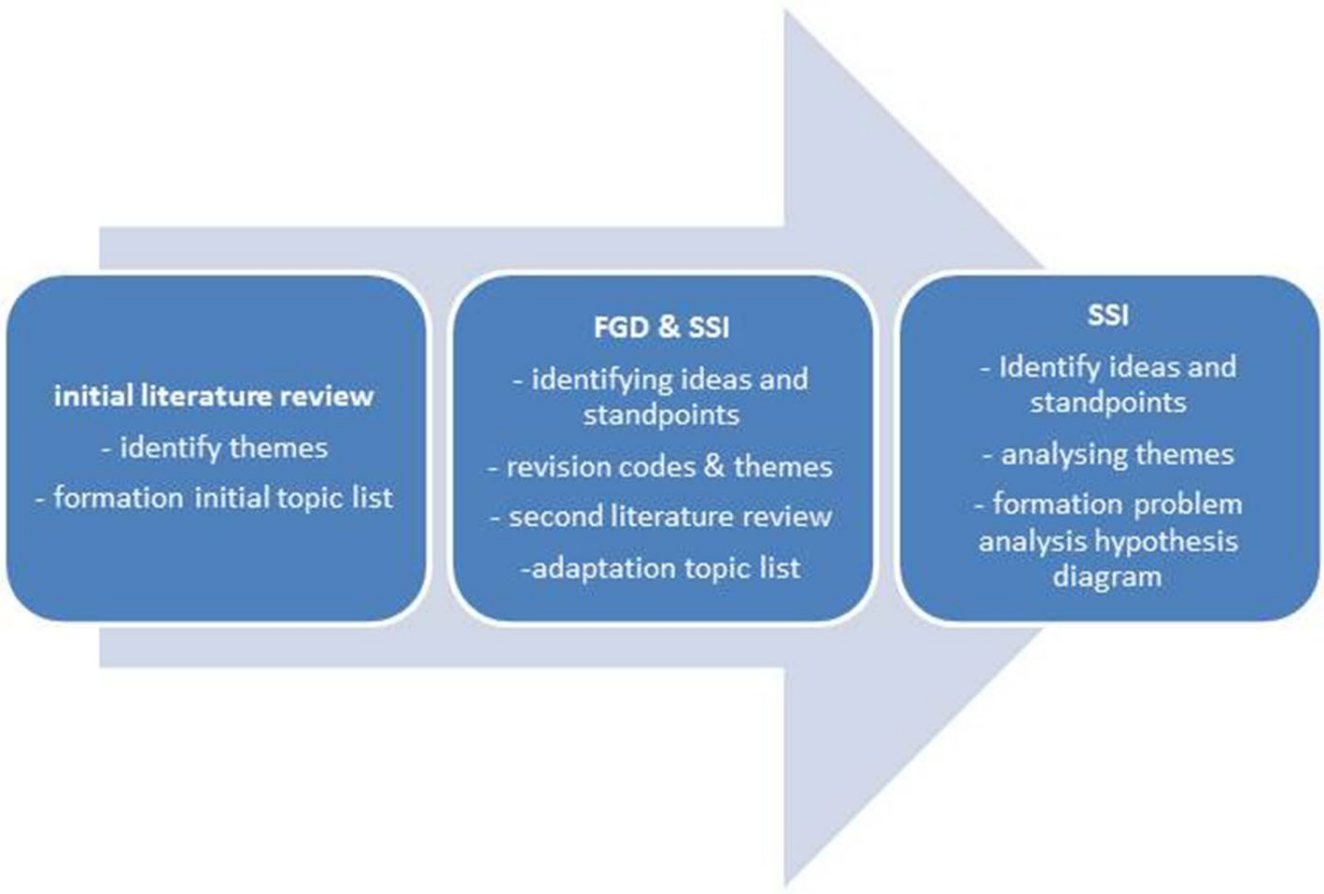
Phases of data collection

**Fig. 4:**
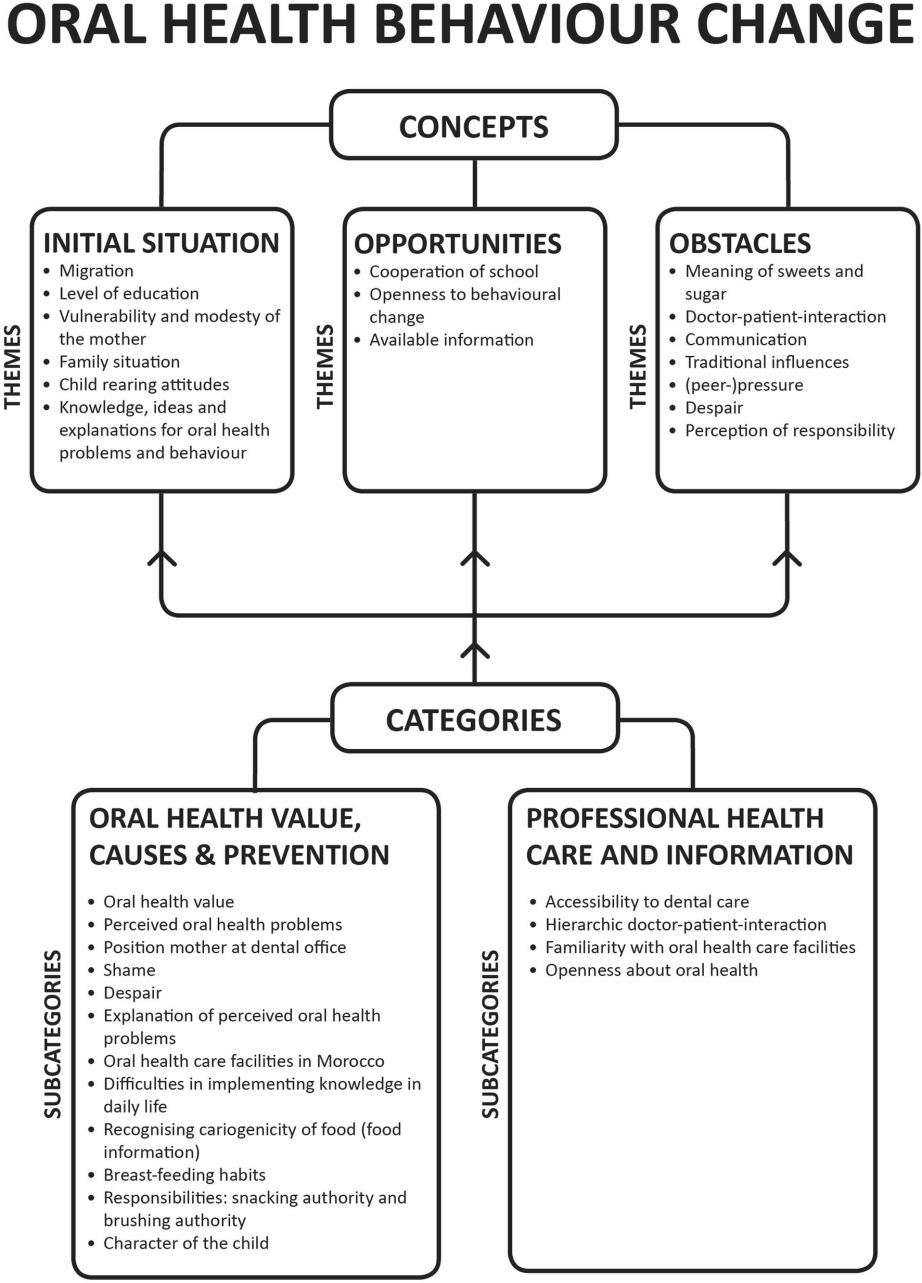
Problem analysis hypothesis diagram. The underlying cultural concepts affecting oral health behaviour change appear in the three constructed main themes identified from the data

The research contained three phases of data collection (Fig. 3) with systematical data analysis (Fig. 1). The first phase was an a priori approach to identify research themes from the literature, which informed the theoretical basis for the initial topic lists. Themes were selected in case they appeared in previous research with comparable aims. In the second phase, these topic lists were used in an inductive approach in two FGD’s (n = 16) with Dutch Moroccan mothers and exploratory SSI’s with three paediatric dentistry professionals and one paediatrician to identified the participants’ standpoints, ideas, and underlying concepts. During analysis, new codes were identified and themes evolved while some initial codes and one theme (association with general health) were removed because they were not reflected in the empirical data. This intermediate analysis was followed by a second literature review on new themes that had emerged in the inductive analysis. This resulted in the adaptation of the topic lists to allow for the acquisition of more in-depth data in subsequent semi-structural interviews with Dutch Moroccan mothers in the third phase of the research. Data saturation was obtained after 13 interviews after which a final literature review completed data collection. Themes and their connections were analysed, resulting in the formation of the problem analysis hypothesis (Fig. 4).

Participants signed an informed consent and completed a small socio-demographic questionnaire. This questionnaire contained questions about age, nationality, cultural group, number and age of children, education of the mother, profession of both parents and household income. The first author moderated all interviews using a topic guide with three probe levels to structure the discussion and to secure all topics were discussed. All FGD and SSI were conducted in Dutch, but an interpreter was required for translation during one FGD and four SSI and the transcription of these interviews. All interviews lasted approximately 1 h and were voice recorded on MP3 and transcribed verbatim. Five bachelor students in dentistry, who received training in qualitative interviewing, assisted in focus group discussions, semi structured interviews, transcriptions and literature review. The students and the first author revised all transcripts. The full transcripts were entered into MAXQDA 10^®^ (Verbi Software, Marburg, Germany, 2010), a software programme for qualitative data analysis, to enable the coding and analysing of the texts. Inductive coding was done independently by the first and last author, and discussed in regular meetings until consensus was reached. A bilingual person translated the quotes from Dutch to English.

## Results

The findings section is structured according to the core themes that emerged from the data analysis, after coding and categorising. The codes were sorted to create (sub) categories, which formed final themes and concepts. In the final part of the analysis the hypothesis was proposed as shown in the theoretical model for analysis (Fig. 4).

The themes can be classified into three main domains affecting oral health behaviour change; namely *initial situation, opportunities* and *obstacles*.

### Initial situation

The domain ‘initial situation’ includes a variety of categories that determine mother’s attitude to oral health care, problems and their causes and prevention, as well as oral health values. It consists of themes rooted in the mothers’ lives: migration, social norms and values; knowledge ideas and explanation for oral health problems and behaviour as well as family situation and child rearing and peer pressure.

#### Migration

Oral health care is used more habitually in the Netherlands than in Morocco. Most mothers mentioned that in many regions in Morocco, dental problems were not considered to be health problems. Some mothers were not aware of existing oral health problems in Morocco, while others mentioned the lack of preventive and restorative care in their region of origin.

(M11): ‘In the Netherlands they have more information, here a child has more knowledge compared to children in Morocco. The child will come home and says ‘the teacher has taught me’ …’

While in Morocco, if there are people that understand or just give attention to the matter, it will happen. Then they will give attention to the child as well. Not in this way, some people have not learned, like they have no knowledge, and then they will just let go.

Mothers believed that their stay in the Netherlands had increased their oral health awareness and their use of oral health care.

(M11): ‘In the Netherlands more attention is given to the children than in Morocco. When you are in Morocco, no dentist will visit schools and check the teeth of children, as happens here. Besides, if you bring your child to a dentist in Morocco, it will never be for check-up. That is more difficult there than in here, because here you will go to the dentist more often…’

Furthermore, mothers mentioned the self-management of children has diminished over the years, even in Morocco:

M5: ‘It used to be different in Morocco. You got up, you got dressed, and you went to school on your own. There were no parents to assist you. Not like here. (…) In the old days, parents helped their children when they were small, but once they attend school, children are independent. My sister has small kids now, she helps them getting dressed, brings them to school, prepares their lunch. This is very different than in the old days, both in Morocco and here’.

#### Social norms and values

All mothers considered a white, healthy set of teeth important for their children although this was supposed to be more important for permanent teeth than for primary teeth:

(M6): ‘They are important, but well, with primary teeth you think, they will shed eventually…’

Mothers did depreciate cavities since these might result in tooth loss. Some mothers indicated black teeth are strong teeth, while most mothers considered black teeth as dirty teeth and seemed to detest the uncleanliness. Mothers believed children less than 4 years old do not have to be exposed to dental preventive visits or were uncertain of the benefit of a dental visit for the child:

(M11): ‘Well the youngest will… I can only take him to the dentist if he attends school [4 years old, **]. He does not have many teeth now, just a few teeth, which he will clean. In case he has a problem, eat sweets or has a problem, I will take him to the dentist.’

(M8): ‘… since I was in doubt all the time, should I take her to the dentist, or…. But everybody told me she is too young, I don’t know if you will receive care because she is still so young, and because she has her milk teeth, that might shed and so on. Then, I just leave it, I thought, maybe that’s true.’

Mothers considered breast milk healthier than formula and regarded the ideal period for breastfeeding to be 2 years. Milk was regarded to be of such importance for the quality of the skeleton, that, when children refused to drink milk from a glass, the mother continued bottle-feeding at will to ensure the milk intake of her child. Mothers observed swelling, pain or black, rotten, and crowded teeth as oral health problems.

#### Knowledge, ideas and explanations for oral health problems

Mothers had a variety of explanations for the oral health problems observed in children. Many mothers mentioned eating sweets and lack of tooth brushing as the main causes. However, they also reported their children having vulnerable teeth and suffering from caries as a family illness, or as the result of taking antibiotics, using a nursing bottle, eating chocolate, drinking sweetened drinks and originating from a country where oral health and dentistry are not incorporated in the health care system. According to most mothers, knowledge about oral health broadened as soon as a mother had more children, which would make her more aware how she should take care of the children’s teeth. However, at the same time, they made it clear that this gained knowledge was not necessarily applied to daily life:

(M1): … maybe I have learnt from the previous…..from my daughter… Yes, maybe… I have heard from the oral hygienist. She said just give food and then cut it off. No more, you should not let the child with that [nursing bottle]… (…) No, the older children did not walk around with the baby bottle. Just after 1.5 years the children have stopped the baby bottle.

Almost all mothers considered reducing sugary snacks and brushing teeth as their main preventive measures for good oral health:

(M2): sweets? ‘I just do not buy it, because they are not allowed to…’

However, it seems mothers had difficulties in recognising the cariogenicity of food and drinks and the frequency of their intake:

(M2): … ‘I buy those raisins, but no sweets, but something they are allowed to have…’ and ‘Well, I mix a bit of Roosvicee [syrup, **] with water, so no juice boxes or uh…. I prefer Roosvicee’,

Supplementary preventive measures mentioned were eating healthy foods and fruits and mothers being in control of their children’s sweet intake, as well as visiting the dentist.

Despite the mother’s knowledge of preventive strategies, hardly any mother mentioned a causal relationship between pain or swelling and a cavity. There seems to be a lack in understanding of the cause-effect relations in oral health and the consequently translation of such insights into practice in daily life.

#### Family situation and childrearing and peer pressure

Mothers had difficulties in implementing preventive measures, for example in supervising tooth brushing at home:

(M1): ‘I try to help him [with tooth brushing, **] I do first, and he, but he wants to do it himself’.

Another mother describes to supervise only the young children:

(M3): ‘If I am not present, he will not brush….No, I do not assist the older children, but the little one. …… he just says ‘finished’, then I will brush his teeth a little bit more…. He [8 years old, **]brushes himself very well’.

Furthermore, mothers also mention difficulties in refusing consumption of juices during school breaks even if they consider juices to be cariogenic:

(M6): ‘Nevertheless, sometimes it is just these juice boxes, apple juice or ……. All the same, he observes it with the other kids and asks me why he cannot have it, while they do. Then it is difficult to explain’

One mother described her struggle in supervising food intake, as the child was growing older:

(M2): ‘it’s only that the older the child, the more difficult it becomes; at home one can refuse, but outside you don’t know. The child can be with a friend who has a bag of chips and yes, you should not really be too strict with that sort of things, because at one point they want to taste and then they want to… you have to offer them, but also you have to prohibit’.

Mothers tend to accept their child’s desire for snacks and to permit it to buy sweets and soft drinks, just as they accept their child’s resistance to tooth brushing. Furthermore, mothers indicated that daily morning chaos and fatigue in the evening kept them performing preventive measures. Mothers mentioned that the quality of tooth brushing and control of sugar intake depended on the character of the child. However, the youngest child in the family was more likely to be pampered by family members:

(M2): ‘I was shocked that my [youngest, **] child had cavities. I thought ‘this young, already’. I was shocked, I thought I am not doing well, or I don’t know. At that time, he was quite spoiled, you know. In the beginning, I did not mind if he would have sweets repeatedly a day. It was only later, when the cavities appeared and he had pain, then I thought, no, it has to quit.’

This demonstrates the lack of self-efficacy in motivating their children to enact preventive oral self-care. Another characteristic of the mother is the vulnerability and modesty of the mother. Mothers demonstrated feelings of embarrassment and shame about dirty black teeth and their children’s oral health problems. Some mothers did not feel comfortable showing their child’s oral health problems at the Children’s Health Clinic (Dutch public infant welfare centre, which provides health care and educational advice to parents of children 0–4 years old).

### Opportunities for oral health behavioural change

Themes like available information and oral health care; and openness to behavioural change and cooperation at school suggest the opportunities for oral health behavioural change.

#### Available information and oral health care

Only few mothers received information about oral health from the Children’s Health Clinic:

(M4): ‘…when the child is 7 months or 6 months, when the teeth…. come out, then, the lady says, you should start tooth brushing… a little bit, because when he drinks milk, it will stay everywhere in his mouth. Even without teeth, one should brush teeth, cleaning the teeth’.

Mothers collected information on oral health at dental offices, although mostly passively.

(M1): ‘My daughter is 10 years old. When she was at preschool, she had such black front teeth. When she speaks or laughs, she is ashamed. She always closes her mouth ‘Look, mommy, I have dirt’. But this was not related to tooth brushing, but because of the nursing bottle when she was little’…’. Yes, she did not want to sleep. She did not want a pacifier. Then I gave her the nursing bottle, then she will drink and maybe she will go to sleep, that’s why (…) ‘I only heard so at the dentist’

Mothers reported their initiative to collect information from the Internet. Most mothers appreciated receiving information at meetings at school or gatherings. They feel at ease in this safe place to discuss subjects with peers.

#### Openness to behavioural change and cooperation at school

Mother indicated they are eager to learn about oral health and are willing to visit information gatherings at schools. They specified their implementing the instructions and adapting their behaviour:

(M2): ‘Yes, we brush in the mornings with the three of us. First, I let them do it themselves, then I will do for them. And, also in the afternoon and evening before going to bed. They know now, they know ‘Oh, we are going to bed, so we are going to brush our teeth’… it really happens automatically now.’

### Obstacles

Finally, themes that demonstrate current obstacles for behavioural change are part of the domain ‘Obstacles’. These include: inferior primary dentition, doctor-patient-interaction, sources of information, meaning of sweets; and fatalism, despair, and perception of responsibility.

#### Inferior primary dentition

Almost all mothers mentioned primary teeth are supposed to be less important to be healthy.

(M8): ‘I do not give her as much sweets as I used to, no. Just because she still had the milk teeth, so…(…) I think, now I have to do it right, because now she will start shedding and after that she will need good teeth.’

(M6): ‘Milk teeth are important, but (…) better to have cavities in your milk teeth, they will shed anyway, than in the permanent teeth.’

#### Doctor-patient-interaction

Some mothers initially consulted the family doctor for oral health problems:

(M3): ‘…just like that, he got up in the morning and had a fat face. He arrived at school and the teacher said ‘No, he should not be in school, he should go to the family doctor’. So (…)I called the family doctor and she said to bring him now, and then she called the dentist. He [the dentist] said to just come to him. So I went there. Luckily they just gave medicine and injection. which helped at that time.’

Mothers expected the dentist to check and clean the teeth and to advise and report to the parents. In some schools, *Jeugdtandverzorging* (school dental service) provides dental care for enrolled children in preschool (4–12 years old). Most mothers considered this service convenient, time saving and of good quality, while some preferred visiting their family dentist, but all mothers requested a dentist who has a child friendly approach. Most mothers preferred to be present at dental treatments.

(M4): ‘with some children they pull out teeth without the parents being present, without permission of the parents… It’s nonsense…Therefore, in any case, if something happens at the dentist mommy or daddy should be present... and they should explain to the parents.’

Mothers expected the dentist to not only give a standard information leaflet but also actively advise about oral health:

(M2): ‘they are open for questions, but only when they see there are dental problems. Then they will raise alarm, but when they observe everything is okay, then they will not mention anything’.

Nevertheless, they complained about the limited time available at the dental visit, which hampers communication with the dentist and processing information given to them.

(FG^1^ M1): ‘the information should be given before, not when the dentist is working on other things and you, as a mother are also busy comforting your child so you cannot record all the information given’.

The dentist was already transferring information to them, while the mothers were still focused on comforting their child in the dental chair.

#### Sources of information

When they observed a problem, some mothers consulted their peers or the Internet, but most mothers indicated oral health to be an issue that is only discussed within their family or with the dentist.

(M1): ‘… we do not talk about the child about the mouth, no… I do not have that many family members here. We just talk with each other, with my husband, my children and my mother… (…) with my sister. Only with our family, that’s it.’

(M2): ‘You have to ask [the school dentist for information] and with my own dentist they explain and advise and tell you if it’s painful for the child. But here [school dentist] no.’

Moreover, lay and traditional ideas about causes of oral health problems can be persistent, even when additional knowledge is acquired. Mothers consulted family doctors or dentists for pain complaints and swelling. They said extraction is the inevitable treatment when pain or swelling is observed.

#### Meaning of sweets

Mothers indicated they are responsible for sweet intake indirectly, as pocket money is used for buying sweets, but more importantly, some mothers are responsible as they used sweets directly to encourage positive behaviour and to comfort the child:

(M9): ‘for the youngest child I do [give sweets, **], for comfort….but for the others, it not for comfort… I used to yes. Sometimes I use it [sweets, **] for comfort…. while there are times I use it as, how to say? ‘Well done! You deserved it’, as a reward, yes. Both actually’.

Besides, some mothers considered sweets to be part of daily life:

(M9): ‘Really, I always have sweets at home. (…) but to my opinion, sweets belong to children. Honestly, I have to say, as a parent, I sometimes forget there are rules. Me, myself I don’t eat many sweets, but sweets should always be present at my place. I don’t know why….’

#### Fatalism, despair and perception of responsibility

Mother had difficulties in sweet restriction of their child. They seemed to hope their child reduces sweets on their own initiative.

(M8): Now she knows, because I told her if you are going to eat sweets again, you will have toothache again. Because she had already had an injection, anaesthesia, and she was really afraid of that. So, honestly, I frighten her a little bit…… So now she takes care, as soon as she takes a sweet, she ask ‘mommy, I do not have cavities do I? it is just like she will, like now I am eating a sweet, so I might get a cavity. But, fortunately she has reduced her sweet intake. So I am happy, yes’

They invented strategies like frighten the children or hiding the sweets at home, however their children continued eating sweets as soon as they found these. It seems mothers consider children eating sweets is beyond their control.

These results are structured and presented graphically in the problem analysis hypothesis diagram (Fig. 4).

## Discussion

This study provided interesting insights into factors that motivate Dutch Moroccan mothers’ preventive care for their childrens’ oral health.

### Initial situation

Oral health care is used more habitually in the Netherland than in Morocco. In general, the duration of stay in the Netherlands influences the level of language proficiency and the level of education, which in turn influences the accessibility of the health care system and what information on oral health is gathered. This might subsequently lead to an improved incorporation of oral health into migrants’ original concept of health. The present study findings confirm that later generations of immigrants, 2nd and 3rd generations, display increased knowledge on preventive strategies, although they indicate the difficulties in its implementation. This finding corresponds to findings from previously mentioned qualitative studies. Aljafari et al. (2014) found that parents of children at high caries risk seem to understand the effect of sugar intake, healthy diet and oral hygiene, but have difficulties in applying this knowledge to daily life; they feel powerless. Mothers mention barriers such as time and a child’s attitude like ‘difficult to brush’ (Miller et al. 2010; Aljafari et al. 2014), and lack of daily routine (Trubey et al. 2014). In the Netherlands, it was also found that routine and structure were important factors to manage tooth brushing twice a day in Dutch children (Duijster et al. 2015a). In line with this, the Dutch Moroccan mothers in our research mentioned the daily morning chaos and fatigue in the evening as barriers to perform proper oral hygiene practices for their children.

In the present study our analysis childrearing values emerged as principal determinants underlying daily life preventive practices. Mothers believe children less than 4 years old do not have to be exposed to dental visits. Moreover, they respect the character and self-control of the child, and do not feel enabled to intervene if such young children resist changing their behaviour. Mothers therefore feel they lack authority regarding the snacking and tooth brushing behaviour of their child. Mothers signal that they feel helpless to motivate the children for tooth brushing. In previous research is was found that parents who categorised their children to be difficult to brush have children with a higher caries rate (Hooley et al. 2012).

It is known from previous research that increased dental knowledge does not necessarily result in oral behaviour change. Recently, oral health literacy was introduced as an overarching determinant to explain lack of improvement of dental behaviour (Hom et al. 2012). Oral health literacy is defined as ‘the degree to which individuals have the capacity to obtain, process and understand basic oral health information and services needed to make appropriate health decisions’. Low oral health literacy is associated with reduced oral health status and lower use of dental services (Divaris et al. 2011) nocturnal feeding and lack of tooth brushing (Vann et al. 2010; Horowitz and Kleinman 2012). The present study results confirm that improving oral health literacy of Dutch Moroccan mothers can positive influence their dental care behaviour. It was found that mother’s attitude and health seeking was influenced by the quality of their knowledge regarding perceived oral health problems and the causes. Firstly, despite the mother’s knowledge of preventive strategies, hardly any mother mentioned a causal relationship between pain or swelling and a cavity. There seems to be a lack in understanding of the cause-effect relations in oral health, which negatively impacts on the translation of knowledge into practice in daily life. Mothers indicated that their dental knowledge increased with every child, which puts them in a better position to provide dental care for their forthcoming children. However, a systematic review revealed that high birth order is associated with higher caries rate and lower level of medical surveillance (Hooley et al. 2012). Secondly, lay ideas about causes of oral health problems can be persistent, even when additional knowledge is acquired. Anthropologist Helman describes that becoming ill is a social process, shaped by cultural factors such as social class, gender, religion and family. In general, Western cultures reckon more factors within the individual as a cause of their illnesses, whereas non-Western cultures indicate more external factors (Helman 2001). Correspondingly, in the Netherlands in a cross-sectional study on parental and family influences, Duijster et al. (2015b) found that parents of Moroccan and Turkish children had significantly more external locus of control compared to parents of Dutch children. In accordance, in the present study many mothers mention causes for oral health problems that can be classified as external factors that indicate a more external locus of control (Duijster et al. 2015a, b).

#### Opportunities

This ‘initial situation’ can be regarded as the foundation for oral health behaviour. Nevertheless; there are opportunities for oral health behaviour change. Firstly, mothers were aware of information being available on the Internet, at the Child Health Clinic, in dental offices, and in school programmes where mothers learn about oral health. It appears that for Moroccan Dutch mothers, school and the Internet are the preferred places to be educated on oral health. If this finding is confirmed in further research future oral health prevention programmes should focus on these information distribution channels. Secondly, the mothers in the present study demonstrated their open mindedness to implement acquired knowledge to behavioural change, by reducing nursing bottle habits, visiting the dentist for preventive reasons, and supervising their children’s tooth brushing. This willingness to implement new information can be regarded as an opportunity for oral health behaviour change.

#### Obstacles

Mothers demonstrated openness to oral health behaviour change; nevertheless the dentist-patient interaction is experienced as an obstacle, since mothers have difficulties in expressing themselves in Dutch and understanding the information they receive from the oral health care professional. This might result in a hierarchic relationship, characterised by embarrassment and shame on the side of the mother. Other obstacles are family structures and values, and household influences. These include: habitually indulging the youngest child and traditional task division of household activities, such as tooth brushing being a mother’s task while they lack sufficient time for adequate hygiene for each individual child. Mothers signalled that they feel helpless to motivate the children for tooth brushing.

A traditional influence is the cultural use of sugary foods to comfort and to celebrate, as well as a disapproval of skinny children. Previous qualitative research on food choices of Dutch Moroccans revealed that social norms prevent diet changes. Hospitality is such an important value to Moroccan families that it results in a higher intake of food than they themselves consider to be healthy (Nicolaou et al. 2009).

A substantial value is the perception of the primary dentition as unimportant. Consequently, less care is taken to maintain this dentition. These findings correspond to those from previous studies (Hilton et al. 2007).

Finally, the perception of oral health as a family affair excludes mothers from new information and experience from her peers. Children’s peer pressure is another obstacle to the desired behaviour. Mothers indicate on the one hand they wish to provide their children healthy school snacks such as fruit and water; on the other hand they do not want to make them uncomfortable by being the only child lacking juices and sugary snacks at school.

#### Strengths and limitations of the study

Considering the qualitative nature of this study, the strength of this research is the focus on cultural factors in researching origin of high caries risk in children. By mapping themes *bottom-up* and in more detail, a profound understanding of the background of classical determinants in caries risk in children can be obtained. As a result, the variant representation of the themes in the problem analysis hypothesis diagram can complement findings and caries risk models from previous studies.

The present study has some potential limitations. First, this research is based on interviews only. The use of participatory observation as a qualitative research method would have yielded a more detailed insight in family habits concerning diet, hygiene and family structures. Unfortunately, this research team did not have the opportunity to implement such a study design. Another potential limitation is that we only interviewed adults. Including children as informants could yield insight in children’s own ideas and strategies regarding their oral health. Anthropologists have advocated this as a technique as children’s perspectives on medical issues are important in their own right, as these inform children’s own health strategies and those of others (Meurs and Van Wolputte 2000). Previously, technique child participatory approach has been successfully applied in research on diabetes (Dedding 2009). For future research it would be advisable to extend the study design and adjust interview techniques to enable including children as informants or co-researchers.

This research included insufficient respondents for statistical analysis and the data therefore cannot be generalised to all Dutch Moroccan mothers. However, this research was explorative and intended to identify possible themes that may impact on mother’s strategies to their children’s oral health and to build a hypothetical model that could be developed and tested in further research. In light of the data saturation reached in our in-depth interviews we are confident that the identified themes reflect the areas that are important in relation to Dutch Moroccan mother’s knowledge, attitude and practices regarding the oral health of their children.

## Conclusion

From this explorative study it can be concluded that Dutch Moroccan mothers appear to have knowledge on preventive oral health practices and oral health literacy, but perceive everyday life barriers limit implementation of this knowledge. In their reflection on barriers mothers mention certain values related to child-raising that have been indicated in previous research to be risk factors for children’s oral health. Therefore apart from socio-economic position, cultural factors, in particular related to child-raising practices, influence children’s oral health and should be further investigated.
